# Development will (try to) find its way: a qualitative study of Chilean adolescent mental health during and after lockdown

**DOI:** 10.1186/s13034-024-00808-x

**Published:** 2024-10-23

**Authors:** Camila Espinoza, Florencia Canessa, Shelley van der Veek, Lenneke Alink, Anja van der Voort

**Affiliations:** 1 Institute of Education and Child Studies, Faculty of Social and Behavioral Sciences, Leiden University, Wassenaarseweg 52, Leiden, The Netherlands; 2 Institute of Psychology, Faculty of Social and Behavioral Sciences, Leiden University, Wassenaarseweg 52, Leiden, The Netherlands

**Keywords:** Pandemic, COVID-19, Coronavirus, Mental health, Wellbeing, Adolescent, Lockdown, School closure, Qualitative, Chile

## Abstract

**Background:**

The COVID-19 pandemic has had a well-evidenced impact on adolescents, who are especially sensitive to pandemic disruptions given the critical role of socialization in their development. In Chile too, evidence shows increases in mental health complaints among adolescents over the lockdown period. Our study aimed at exploring the experiences of Chilean adolescents regarding their mental health during the lockdown and school closure (March 2020-December 2021), and during the return to on-site education (2022) as informed by adolescents and school staff, with a focus on family, school, and social sources of risk and support for adolescents’ wellbeing during these periods.

**Methods:**

Using a qualitative approach, we conducted semi-structured interviews with 19 adolescents and 16 staff members from schools in an urban area of Chile.

**Results:**

Through thematic analysis, we generated five themes: [1] *Adolescents in a mental health crisis*, comprising a range of distressing experiences and mental health problems. This crisis was fueled by alterations in the functioning of adolescents’ systems: [2] *Broken support systems* (peers and school); [3] *The school agenda must go on*, reflecting schools’ strict compliance with the educational curriculum; and [4] *Blurred boundaries* between home and school life and within the family. Finally [5], *Development will (try to) find its way* describes how most participants experienced a bouncing back to wellbeing in the course of the school year upon return, and how some developmental milestones took place despite the abnormal conditions, providing evidence for resilience amid pandemic adversity.

**Conclusions:**

The findings give insight into how the exchanges between the adolescent and the social systems they are embedded in were interfered. The results help us understand the challenges for mental health during and after the pandemic, and highlight adolescents’ capacity to thrive as normality was restored. The results also underscore the importance of upholding stability across adolescents’ systems and routines, in order to mitigate impacts on wellbeing amid abnormal circumstances. The findings are relevant for development-informed initiatives in policy design in the aftermath of the pandemic and in future crisis management responses.

## Introduction

Throughout the world, the COVID-19 pandemic had a negative impact on the mental health of people of all ages. Particularly severe effects have been found in vulnerable populations such as adolescents [1], whose development is especially sensitive to the effects of social deprivation. In regions with harsh pandemic control measures (e.g., long and stringent lockdowns), the effects of COVID-19 on adolescents’ development probably were and still are the most concerning [2–4]. While COVID-19 is currently no longer considered a health crisis, the need to document mental health sequelae remains relevant. This study focuses on the impact of the COVID-19 pandemic on adolescents in Chile, where teens and their families lived under lockdown for almost two years, disrupting social life and normal routines, including schooling. Our aim was to deepen understanding of the impact of the pandemic experience -particularly the lockdown- on adolescent mental health, and explore processes in adolescents’ family and school environments that supported adaptation during the pandemic and enhanced functioning afterwards.

Since adolescence is a sensitive period for the onset of psychopathology [5], it is vital to study which factors increased the risk of adolescents developing mental health problems amid the pandemic. In a life stage when autonomy and social experiences outside the family system are essential for normative socioemotional development, the consequences of pandemic restrictions might pose unsuspected mental health challenges. The pandemic control measures disrupted adolescents’ opportunities for socialization, which in addition to the chronic stress of the pandemic could have pervasive effects on emotion regulation [6] and the developing brain [7, 8]. Besides friendships being a key buffering factor for individual resilience [9], socialization with peers aids the maturation of executive functions and social behavior [10], learning [11], emotion regulation [12], and identity development [13].

An ample body of literature demonstrates a deterioration of mental health among young people during the pandemic, as compared to pre-pandemic levels [14–16]. Globally, social distancing and lockdowns were associated with decreased quality of mental health, reflected in a rise in internalizing and externalizing problems [11], psychiatric emergencies [17], and suicidal behavior [18]. The risk of internalizing and externalizing problems was increased by cumulative exposure to pandemic-related stressors [19]. In some studies, mental health complaints were also related to exposure to specific pandemic stressors such as school closure [20], daily life disruption [21], social isolation [22], online learning, and interruption of contact with friends [23].

### The Chilean case

The pandemic arrived in Chile at the beginning of the school year (March 2020), amid a deep socioeconomic crisis. Soon after the first positive cases, the government implemented a dynamic lockdown (“Paso a Paso” or “Step-by-step” plan), in which confinement measures were periodically tightened or loosened in different locations, depending on infection rates and population health risks [24]. In practice, for most of the population the Step-by-step plan meant that for almost two years daily life was unstable and uncertain. For school-aged children particularly, school closure policies amounted to an inflexible educational regime, out of tune with young people’s developmental needs.

In an attempt to continue with the educational agenda as instructed by the national curriculum, schools tried to adjust the delivery of education as much as possible, making a major effort to quickly transition to digital remote learning upon closure [25]. The Ministry of Education provided schools with financial aid for improving infrastructure (in a way that could comply with safety protocols), student connectivity, and food provision for the most vulnerable families. In October 2020, the Ministry of Education advised in-person education if schools could comply with safety measures, but only a small percentage of them had the means to do so (e.g., meeting the space requirements to allow all children to attend with distancing) [26]. Nearly half of schools were still exclusively teaching through online synchronic classes in the first semester of 2021 [27]. Towards the second semester of 2021, on-site educational activities slowly became more feasible in compliance with the remaining disease-control measures. However, in practice this meant that not all classmates could attend at the same time because of the limited indoor capacity, or even that whole classes had to quarantine after close contact with infected classmates. At this point, attendance remained optional, with the result that students’ daily routine and peer connections continued to be unstable. On-site education and mandatory attendance were only reestablished in full from March 2022 [28]. Students had to readapt to the educational system with several measures still in place, such as masks, access restriction based on vaccination, and limits on the numbers allowed inside classrooms. It was not until October 2022 that face masks were no longer mandatory everywhere, meaning that students could resume their free socialization in and outside the school setting.

In light of this situation, of all countries in the OECD (Organization for Economic Co-Operation and Development), Chile is the one with the most days of full school closure [29], although the restrictions did not affect all schools in the country evenly [25]. The high social inequity of the country was reflected in the conditions for education that schools were able to provide: for students from public schools, access to online classes was more likely to be limited, and these schools had little capacity to adapt infrastructure to the social restrictions. Retrospectively, reopening decisions can be seen to have been highly dependent on the type of school funding, in large favor of private schools attended by the most economically advantaged students [27]. Moreover, during this period school dropout and severe absenteeism increased substantially compared to pre-pandemic levels and, as in other countries, children presented significant learning lags on their return to on-site schooling [30]. Due to digital and economical barriers, children from families under higher financial strain were also disproportionately affected by the education system implemented during the pandemic [25]. They were also probably the most impaired in their emotional wellbeing [31], since disadvantaged groups were more exposed to pandemic-related hardships [32].

The prevalence of psychiatric disorders among Chilean young people was already high before the pandemic (38.2%) [33], with studies showing increasing trends of anxiety, depression, and behavioral problems for this population [34]. The pandemic is thought to have raised these numbers even further. When schools reopened, public opinion spoke of an adolescent mental health crisis, with warnings about increased violence between students and from students against their establishments [35]. Despite these concerns, peer-reviewed publications on Chilean adolescents’ mental health during and after the pandemic are scarce. The few studies available show that Chilean adolescents’ mental health declined during the pandemic compared to pre-pandemic levels [31, 35, 36], and adolescents in Chile scored higher on depression and anxiety during the pandemic than those in other Ibero-American countries [37]. Little research has looked into the extent to which the disruption of school, socialization, and daily life impaired wellbeing during adolescence in countries with severe restrictions like Chile, or identified features that might have aided adolescents’ adaptation in the transition to normal life.

Studying the development of adolescents in Chile during and after COVID-19 is not only useful at the national level, but also sheds light on the development of adolescents in such crises more broadly. Worldwide, the emerging literature on the COVID-19 crisis has focused on identifying the prevalence of mental health problems and their risk/protective factors. However, most studies fail to explore in depth *how* the experience during the pandemic may have led to mental health impairment, and what individual or social factors may have aided adolescents’ resilience to navigate the pandemic period and the subsequent transition to “normal” life after the pandemic. Moreover, studies have rarely addressed specific groups such as adolescents, especially during the transition period *after* the strict confinement measures. Further, most of the existing literature on predictors of mental health– during and in the wake of the pandemic- comes from studies conducted in highly developed countries [38], leaving most Latin American countries (with the exception of Brazil) outside the scope of research [39, 40]. Latin American countries faced particular structural challenges such as socio-economic disparities and high prevalence of mental health issues, which made them ill-equipped to buffer the psychosocial impact of the pandemic [40]. Moreover, only a handful of studies [41–43] have qualitatively explored the impact of the pandemic on adolescents using in-depth methodologies that help understand adolescents’ adaptation processes in the course of the pandemic and the unique challenges they had to face. To our knowledge, no qualitative studies have explored factors contributing to adolescent wellbeing in the post-pandemic period.

### The current study

Using an in-depth and exploratory qualitative approach, our aim was to address the impact of the pandemic experience on adolescent mental health, from the perspective of adolescents and school staff. We particularly focused on the effects of the two years of home schooling, by collecting retrospective perceptions of adolescents concerning their mental health. Additionally, we explored features of the family, peer group, and school setting that, according to adolescents, were protective or burdensome for their wellbeing during the lockdown and on their return to on-site schooling. We also collected school staff members’ perceptions concerning adolescents’ mental health during these challenging times, and explored which factors they perceived to be of influence on that mental health. This work aims to make visible the challenges in adolescents’ mental health during and after the pandemic, in a region that usually falls outside the scope of the mainstream scientific literature, highlighting priorities for prevention and intervention after the crisis.

## Methods

### Study design

This paper reports on a qualitative study that was part of a larger project that also included a quantitative study aimed at examining the mental health of adolescents in the transition to face-to-face education after two years of school closure. The study complies with the Consolidated Criteria for Reporting Qualitative Research (COREQ) guidelines for reporting qualitative research [44]. Using semi-structured interviews, we aimed to collect the views of adolescents and school staff regarding the impact of the pandemic on adolescents’ wellbeing.

### Participant selection

For the recruitment, we attempted to contact all of the public, partially subsidized and private high schools listed in official records of an urban area in Central Chile (*k* = 70). Of these, we were able to make actual contact with 49 schools, while attempts were unsuccessful with 21 schools (e.g. phone lines out of service or non-responsive). The schools would be invited to take part in the full research project, comprising both the quantitative stage and subsequent qualitative stage. Of the 49 schools initially contacted, four (8.2%) agreed to participate in the study, while 20 schools (40.8%) actively declined and 25 (51%) did not respond further. Participating schools varied in institution size, type of financial basis (public schools, fully subsidized by the government, and semi-private schools that receive some funding from the government and some from third parties), and vulnerability index (see Table 1 for an overview). Consequently, the participating students varied in socioeconomic characteristics.

**Table 1:** Participant school characteristics

ID	Size^a^	Funding	School type	Vulnerability index^b^	Participants: Staff (*n*)	Participants: Student (*n*)
School 1	Small	Subsidized private	Elementary/ high school	> 85%-<90%	2	6
School 2	Medium	Subsidized private	Elementary/ high school	> 80%-<85%	4	4
School 3	Small	Subsidized private	High School	> 90%-<95%	5	3
School 4	Large	Public	Elementary/ high school	> 75%-<80%	5	6
Total					16	19

^a^Students enrolled in high school in 2022. Small: 1-100 students; Medium: 101–500 students; Large: more than 500 students

^b^Percentage of low-SES students enrolled in high-school classrooms (1st to 4th grade) in 2023

We recruited adolescents to participate in the larger study through digital and paper invitations delivered by the school administration. In order to participate, adolescents first had to share the invitation with their parents to discuss participation and obtain parental consent. The inclusion criteria for the larger study were enrollment in first, second, or third grade of high school (students’ age: 14 to 17 years) and having the parental consent form signed. For the interview part, we selected 19 participants from the group who took part in the first quantitative study (*N* = 419), who also signed up for the interview (*n* = 191). We used a purposive sampling strategy: for each participating school, we randomly selected an even number of girls and boys per classroom, of students per grade level, and of number of students from the larger pool of enrolled volunteers per school. We intended the group to be as diverse but also balanced as possible in terms of classroom level, gender and school. Differences in balance are related to the actual interview enrollment per school. For an overview of the participants see Table 2.

School staff were invited to enroll for the staff interview via a QR code on the flyer they received from the researchers. The inclusion criterion for the school staff was having a direct relation with the target classrooms (1st to 3rd grade of high school) as part of their job. Since it was not possible to recruit an equal number of staff with different roles from the four schools, the final composition depended on voluntary enrollment at the time of data collection (convenience sampling). The final group comprised 16 staff members from the four schools (see Table 2). All participants received a gift card to the value of $5,000 Chilean pesos (approximately 5 euros) for their time and contribution.

**Table 2:** Overview of participants

Adolescent group		Staff group	
*N*	19		16
Gender			
Male	11		7
Female	8		9
Classroom		Role	
1st Grade	5	Administrative	2
2nd Grade	9	Main teacher	4
3rd Grade	5	Subject teacher	10
		Learning support^a^	2
		Psychosocial support^b^	3

^a^e.g., remedial teacher

^b^ e.g., social worker, psychologist.

### Data collection

Individual interviews took place in November and December 2022, before the end of the school year in Chile, soon after the government ended the mandatory use of face masks in schools and the indoor capacity restrictions. The interview guides were created by CE, SV, and LA, and translated into Spanish by CE. The guide for adolescents comprised questions regarding their overall experience of the stay-at-home period; their emotional states; changes in family, home life, and friendships; their experience of the return to face-to-face school; and their perceptions of support within the family, peer, and school setting. The staff interview addressed the interviewee’s perceptions of adolescent mental health during the lockdown; changes observed in adolescents’ mental health on the return to on-site education and throughout the school year; support strategies provided to students by the school; and continuing challenges for adolescent well-being in the post-pandemic period.

The interviews were conducted in (Chilean) Spanish by three local qualified clinicians (including CE), in a suitable private facility in the school setting, or–for school staff– via video call if preferred (4 participants). All interviews were audio recorded upon consent.

The audio recordings were transcribed verbatim by a native speaker of Chilean Spanish. The quality of the transcription was checked by CE, and interviews were anonymized by FC. We found high variability in the length of reports about the pandemic experiences of adolescents (interview length range = 11–55 min, *M* = 21.5 min) and school staff (interview length range = 12–84 min, *M* = 35.8 min). The quality of the interview content was discussed among FC, CE, and AV. Differences in interview quality could not be attributed to methodological issues (e.g., the role of the interviewer and contextual factors). Shorter interviews were due not only to some participants giving less elaborate answers, but also to some of them having nothing to report in response to certain questions (e.g., adolescents who did not experience changes in family dynamics, or staff who did not work at the participating school during the pandemic and therefore could not report on the mental health of students during lockdown). Therefore, all interviews were included in the analysis. Quotations supporting the analyses were translated from Spanish to English by FC and CE.

### Analytic strategy

To address the research aim regarding the mechanisms underlying mental health experiences, our analysis followed a critical realistic approach [45]. We conducted a thematic analysis [46, 47], with some deductive codes based on theory, and mostly inductive codes based on the content of the interviews. Coding was performed with ATLAS.ti 23 software [48]. The coding for each group of participating staff and adolescents was done separately, but followed the same iterative steps: First, two coders (CE and FC) read the transcripts to familiarize themselves with the corpus of data. Second, the coders performed joint coding of a set of 6 interviews to elaborate a first set of codes. Third, they evaluated the codes independently to review discrepancy and fit of codes and quotations. New codes and deletions of irrelevant codes were made by reaching consensus between both coders. Based on this first joint coding, a code list was created, which guided further coding. The remaining interviews were split between the two coders, who coded them independently. The new codes that emerged in this single-code round were discussed, reviewed backwards against the double-coded interviews, and subsequently reviewed by each coder throughout the single-coded interviews assigned to the other coder. Analytic memos were registered to inform later analyses. The final codebook was again revised by both coders, and discussed to reach consensus. A set of 73 codes was generated from the interviews with adolescents, and a set of 62 codes from the interviews with school staff. The final list of codes was grouped into higher-order categories and used to identify initial overarching themes. Codes were created at a manifest level, while themes were identified at a more latent level [46]. The authors held iterative discussions on connections between categories and the narratives, uniqueness, and consistency of the themes, as well as on possible overlap with other themes.

### Ethical considerations and reflexivity

This project was approved by the Ethics Committee of the Institute of Education and Child Studies, Leiden University, dossier number ECPW-2022-360. School staff provided their consent to participate and be recorded, and for their answers to be used anonymously in the study. In the case of the adolescent interviews, only adolescents who agreed and whose parents had given their consent for them could participate in the interviews. Adolescents were asked to provide their assent before starting the interview, and agreed to audio recording. The privacy of participants was preserved at all times (e.g., safeguarding privacy of the content of the interview by conducting it in a facility that allowed confidentiality, and ensuring data protection measures regarding personal data collected).

At the time of the study, adolescents were fully back to schooling on site, and all restrictions had been dropped, allowing for a reasonable timeframe for the adolescents’ adjustment, which minimized the risk of emotional harm from the content of the interview. Participating adolescents also received a brochure with information about free mental health services at the end of the interview.

CE, FC, and the interviewers are native locals, fluent in the participants’ language and trained in developmental psychology. FC experienced the pandemic in the same country as the participants and worked as a psychologist with school-aged children, while CE, SV, LA, and AV experienced the pandemic in another region with less stringent safety measures. CE was on site and participated in data-collection activities. SV, LA, and AV are senior researchers in child and family studies, and experienced the pandemic with school-aged children in a different culture. AV has extensive experience in qualitative research. The authors and interviewers had no existing relationship with the participants or with the schools enrolled in the project.

## Results

In the thematic analysis, we generated five main themes that represent the adolescents’ experiences during the COVID-19 pandemic (see Table 3 for an overview). The first theme, “Adolescent mental health in crisis”, depicts the range of emotional and behavioral difficulties that adolescents experienced during the lockdown and upon their return to school. The second theme, “Broken support systems”, describes the extent to which adolescents perceived a lack of support within family, peer, and school systems during lockdown. The third theme, “The school agenda must go on”, reflects the strategies to continue schooling despite the feasibility issues and learning challenges identified over time. The fourth theme, “Blurred boundaries”, identifies the functional changes in the transactions between family and school systems, and the reorganization of the family system during daily life at home as a result of lockdown measures. The fifth theme, “Development will (try to) find its way”, describes developmental processes that took place in the social and individual domain, despite the pandemic. Overall, themes 1 and 5 describe individual outcomes and experiences in relation to adolescents’ emotional wellbeing, while themes 2, 3, and 4 identify risk factors in the ecological context of adolescents that might help us understand the negative mental health outcomes experienced by adolescents.

**Table 3:** Overview of themes and subthemes emerged in thematic analysis

Theme	Subtheme
1. Adolescents in a mental health crisis	Reports of a crisis
	Trying to cope with the crisis
	The crisis beyond the lockdown
2. Broken support systems	The school system
	The peer relationship system
	The family system
3. School agenda must go on	
4. Blurred boundaries	Blurred boundaries between school and home life
	Blurred boundaries between school and the family system
	Blurred boundaries within the family system
5. Development will (try to) find its way	Social domain
	Individual domain: personal development

### Adolescents in a mental health crisis

Adolescents made clear reports on their mental health during lockdown: how the lockdown affected their mental health, and the various strategies they used to cope with these complaints. This emotional toll became evident to school staff after the return to on-site education, when adolescents’ emotional struggles were manifested in the school setting.

#### Reports of a crisis

Adolescents reported on how their mental health was affected during lockdown through a wide range of complaints. Most of them described signs of emotional internalizing complaints, like mood- and anxiety-related difficulties. For instance, many adolescents reported feelings of sadness and discouragement: “Something like stifled, like alone, sad” (A. 16); “I had a lot of sadness. From May until August, I felt very listless, I could not get out of bed... I was tired all day” (A. 14). Feelings of sadness were sometimes accompanied by feelings of anxiety, triggered by daily life during the pandemic, like being locked down or having homework piling up. A large proportion of the adolescents interviewed described feeling emotionally distressed, unstable, or irritable.

Adolescents also reported feeling bored, or experiencing a lack of will power to do things: “In the beginning, it was cool... then, going back to the same routine, being locked down, seeing your family every day, with nothing to do– it became too boring” (A. 13). The feelings of lack of will often co-existed with an overuse of screens and a reduction of physical activity: “It was like… I did nothing but sit at a computer... I did not do any other activity” (A. 16); “I was like looking forward to doing sports and all, but no. I didn’t do it. It’s because I was watching too many series... and then said ‘okay, tomorrow’, and in the end I never did [sports]” (A. 8). On a similar note, adolescents themselves reported the emotional impact of physical changes that might have happened normatively or may have been due to the decreased physical activity during the lockdown:

I got overweight... I was always a person that moved a lot; I did lots of things, trained almost every day; and let´s say, my mind started to turn against me. I said ‘no, it’s not necessary for me to go out, so nobody will see me’. (A. 19)

In some instances, participants’ emotional states seemed to affect their physical wellbeing in other pervasive ways, like alterations in their sleep patterns (insomnia, excessive sleeping, disruptions to bedtime schedules) and eating behaviors:

Many sleep problems: sometimes I could go more than 24 h without sleeping... and I also had trouble with eating. It was like, sometimes I got up, ate food all day, like a binge… and then didn’t eat anything else until the next day. (A. 14)

Among the most common feelings reported by adolescents were loneliness and isolation. In some participants, isolation was described not so much as a consequence of the lockdown, but as a drive to socially “shut down” (A.19) or emotionally withdraw in a way that can be understood as an internalizing manifestation:

I would place myself like in a bubble, just staying in my bedroom, not doing anything. That sort of suffocated me... I would start crying and keep everything to myself... I would talk to myself. (A. 16)

For some participants, anxiety accompanied their drive to emotionally or socially distance themselves. They experienced anxious feelings about socializing -even digitally- and were afraid of being exposed to others during lockdown. For these students, the online schooling experience was a mental challenge and not a welcoming event:

[I felt] constant fear of being chosen to write [in the chat during an online class]... or to read or answer something; when they checked the attendance, I could not say ‘present’: I wrote it in the chat because I felt embarrassed. (A. 17)

Other psychological problems mentioned were somatic complaints, not explained by underlying medical conditions, and attributed by the adolescents to the excessive use of computer screens during remote education: “I had to leave [the online class] because my head hurt after being in front of the screen for so long” (A.19). More pervasive symptomatology in reaction to stress was also reported, such as difficulties with impulse control (e.g., hair or lip-pulling).

Although most consequences reported by adolescents were negative, a few adolescents reported some initial positive reactions in response to the pandemic: “In the beginning, I was relaxed... I prefer staying home” (A.14). A few adolescents even mentioned a sense of normality, referring to feeling “normal” in an evidently abnormal situation: “Eh, no, nothing. Like, normal, as though I was at school” (A. 4). These references to normality were often made by participants who, despite reporting some form of distress, had only brief narratives about their lockdown experiences and struggles. Overall, adolescents reported many more negative aspects of the lockdown than positive aspects.

#### Trying to cope with the crisis

Adolescents not only gave indications of a mental health crisis, but also reported distinct strategies to cope with this crisis. About half of them reported that it had helped them to express their feelings to others and share experiences, mostly with peers. Other adolescents reported that going out and maintaining some structure in their daily activities helped them to remain stable and cope with the difficulties. Interestingly, another strategy that was commonly referred to was isolation. While some adolescents mentioned isolation as a manifestation of internalizing behavior and feelings of distress, others used isolation as a coping mechanism. For example, isolation was useful for some adolescents as a way to protect themselves from conflicts in their home environment and to “ignore” their surroundings (A.9). Adolescents also referred to isolating for recreational purposes, seeking calmness by becoming absorbed in hobbies like music or art: “I hid myself inside, in music, as though to distract myself. In the nights… Like, turning on some music, reading a bit, and well, watching anime helped a lot. But the key thing was music” (A. 19).

Other adolescents referred to digital media and other hobbies as regulatory strategies to counter the exhaustion of online classes. Because in some cases school attendance was reduced to merely logging in to the class, some even chose to do these activities parallel to attending classes: “I stopped watching the classes– I mean, I played it, but only turned the volume up and would then do something else, only listening to the class while drawing or playing and stuff” (A. 2).

#### The crisis beyond the lockdown

The mental health crisis that was reported by adolescents was not restricted to the lockdown period. Upon the restart of face-to-face schooling in March 2022, teachers observed their students’ expressions of unrest, often reporting concerning behaviors (e.g., aggression, bringing weapons into school, suicide attempts and self-harming, sexual activity inside school, or damaging school property) or acute emotional difficulties (e.g., panic attacks, crying during school hours): “The number of kids having panic attacks is huge. Kids are doing horribly, having crises during exams as never before. They keep on crying and all that stuff” (S. 2).

Staff also reported repeatedly about students displaying behaviors related to difficulties in re-engaging and adjusting to the classroom setting, such as difficulty interacting with others, sudden need to leave the classroom, high levels of conflict and reactivity among peers and with school staff, resistance to school rules, and intermittent attendance:

The psychologists were outnumbered by the cases. And we had very extreme cases: students with suicide risk, cutting themselves... They popped up like never before, a lot of cases of students that endangered their own safety... We saw students hitting each other in front of teachers... I even received punches when separating students. (S. 16)

According to school staff, the interruption of normative socialization had negative effects on students’ self-concept. Some staff described adolescents struggling with their self-image and being anxious about socializing on the return to school: “Kids are experiencing a lot of shame, until now, facing some stuff. For example, for many, it is frightening to present something on the whiteboard. Something that for us was super normal, for them seems impossible” (S. 7).

Participants from both groups made references to the effects of face masks on several domains of development. They mentioned that masks interfered with the interaction with peers, because they were “not able to read full body language” (A. 19). Staff also reported on the negative effect that masks had on the delivery of educational activities (e.g., in relation to emotional education). Face masks were also mentioned as an interfering element when it came to the development of self-esteem and social skills. Students sometimes even used the mask as a way to deal with uncomfortable normative physical changes and cover up insecurity issues:

I think it is particular cases. There is one student with severe acne– when they want to stay hidden, they use their hoodie and mask. I have a student who I am sure must be depressed, also with a hoodie and mask. I have never seen their face... And someone I talked to about it said they felt insecure, but I think it was something with their teeth. Then they wanted to continue using their mask... in order to cope better they felt protected behind the mask. (S. 9)

Because not all schools in Chile offer secondary education, half of the participants reported a change of school (transition to high school) right at the start of or during the pandemic. For these students, not having prior social connections with their class was an extra challenge during the pandemic and on their return to on-site schooling. According to staff, during the period of voluntary on-site attendance, new students tended to attend less often than students who were already enrolled at the school and showed more reluctance to interact with their peers in the classroom:

In order to interact, children first need to feel comfortable where they are, in the classroom with their classmates, and they did not know each other... they were with strangers in the classroom. It was like they had just started the first day of first year. (S. 16)

Overall, the adolescents’ own narratives and the reports by staff about their students’ emotional state on their return to face-to-face education seem to complement each other; together they provide a more complete picture of the adolescents’ mental health struggles, which are likely attributable to the pandemic disruptions.

### Broken support systems

Under normal circumstances, adolescents typically turn towards peers as their main source of social support. The school setting would usually also provide a supportive structure and serve as a monitoring agent for adolescent wellbeing. Given the pandemic restrictions, both these support contexts were disrupted for a prolonged period, leaving the family as the most available source of support.

#### The school system

Since the pandemic disrupted contact between school and students, staff could no longer monitor students’ mental health. Many staff members reported that their school had little or no contact with students during the pandemic period. This impeded the school’s usual role in signaling emotional difficulties, which is largely dependent on the students being in the school setting, and the staff being available as significant figures of support for adolescents. One reason for the lack of connection may be that students were reluctant to reach out to their teachers online and communicate their need for support: “The kids are not very good at telling us about their issues in a virtual way… so it is only in 2022, this year, that I have found out more about their struggles” (S. 7). Only a few adolescent participants mentioned seeking or receiving support from school during lockdown– mainly through the school psychologists who were more likely available for emotional support as part of their role. Teaching staff pointed out that being worried about academic work prevented them from focusing on contact with students: “Suddenly, you would think ‘it’s been a month since that student disappeared’. And you would be worried about grading– ‘How will I grade her?’– rather than worrying about why she’d been missing for a month” (S.16). A few staff members reported that the school only implemented psychosocial monitoring when they lost contact with students completely– those that clearly did not show any sign of attending online classes– and it was only possible in a very few cases to make referrals to specialized care if more help was required.

Overall, there seemed to be a consensus among most staff that there were not enough resources to deal with the mental health crises of their students on the return to on-site education. Some staff reported feeling limited in their role of supporting students and needing additional tools and training for this:

There is no way that we can perform in fields that are not our own, and we are not associated with any mental health practice or whatever... [i.e., during a panic attack] where do I send them? To the nurse? The nurse? The woman comforts them, looks at them… what else can she do? She then takes their blood pressure, their temperature, and calls home– their mum or dad– to come and pick them up. Because that’s where our role ends. (S. 2)

Many staff members recognized that their school had not implemented actions to support students emotionally during the pandemic. Only a few individual initiatives were taken to provide emotional support for students on their return to on-site education. Concretely, staff reported being available to talk to students if they were approached. However, this support seemed to represent an extra effort from their side, something that “comes from within” (S.2) rather than a role they were qualified to perform or instructed by the school to take on.

Adolescents who entered school just before or during the pandemic had no sense of belonging to the institution or bonds with it or their peers on returning to on-site education. This was an additional challenge, as staff had to invest substantially more effort in connecting with them in order to play an educational and supportive role:

I had a class for two years during the pandemic, and now I met them live, and we were strangers to each other... I could barely recognize even a single voice... And as they never turned on camera, I didn’t even know their names... Sometimes one of them asks me ‘teacher, how do you envisage me in the future? Do you think I would be good at [field]?’, and another student says ‘Why would you ask them if they don’t even know us?’ (S. 16).

School staff also stressed the shortage of staff qualified to handle mental health in schools. Some schools did not have a psychologist, or if there was one available, the demands of so many adolescents in need of care exceeded their capacity. A few staff members even had to deal with reports of suspected abuse and neglect in the students’ family environment. In more severe cases, where they needed to refer a child to professional healthcare providers, the networks were also not working optimally during and after COVID-19: “The system has collapsed... Many students are on a waiting list in very complex situations, not like just having a panic attack” (S. 17). As a result, many students did not receive the help they needed, either from the school or from the specialized institutions that are supposed to deliver such services.

#### The peer relationship system

Restrictive confinement in Chile naturally disrupted adolescents’ opportunities for socialization. Since the school setting is the main platform for peer interaction during adolescence, friendships with peers were clearly reported as a support system at stake during the pandemic. Most adolescents identified the restrictions on meeting and hanging out with their friends as a significant struggle. Some adolescents dealt with this struggle by not obeying the rules. They mentioned that they continued to meet people in person despite the recommended measures. Clearly, for many it was difficult to overcome the feeling of loneliness by reaching out to others through alternatives to in-person interaction: “It changed a lot: I could not see them [friends], I didn’t talk to them; I spent the time alone, without talking to anyone” (A. 10). In a few cases, contextual factors (e.g., moving to a different city, living away from friends, parents not allowing contact with people outside the household) posed extra difficulties for attempting any contact with friends. In some cases, friendships did not survive the distancing, and adolescents referred to actually losing friends: “We went from meeting up all the time to not knowing anything about anyone” (A. 13).

#### The family system

Given that during lockdown adolescents spent most of their time within the family environment, we might expect to identify family as an important source of support. However, while many participants referred to their parents as a source of support during the confinement, only a few mentioned concrete actions by parents (or older siblings). For example, some parents activated external sources of support when their children showed mental health or learning issues, and others were emotionally supportive:

My mum was always there... Perhaps she didn’t have ideal advice, or very clear answers, but [she] always tried to guide me with her experience, and find a way that, how to say it… would fulfill me more (A. 19).

In conclusion, the lack of physical proximity to peers, the diminished support provided by the school system, and the pressures on the family unit collectively contributed to an inadequate support network for adolescents, which may have fueled the mental health issues previously mentioned.

### The school agenda must go on

Apparently, the educational system continued with its program as normally as possible: “We did the same [online, as if they were at school]. We tried to replicate [the classroom], but in front of a screen, to keep on going” (S. 18). Some adolescents indicated that online education was somewhat more convenient than studying at school on site. However, this convenience was mostly associated with practical benefits, such as not having to wear school uniform, having extra time for leisure activities, not having to commute to school, staying warm during bad weather, waking up later than usual, being able to do everything from their bedroom, or having more relaxed mealtimes.

Taking online classes is the most salient experience that adolescents recalled over the two-year period of lockdown, with most students perceiving it as exhausting: “It was a bit enslaving, having online classes, because it did not leave space for doing different activities” (A. 19). Moreover, studying at home clearly had its downsides, since conditions were not ideal (e.g., attending online classes in the face of connectivity issues).

When teachers and schools tried to come up with an online school schedule, students tried to adjust by creating their individual routine. This routine varied across adolescents, from very structured to completely unstructured or even “bad” (A.17). In addition, the routines differed depending on solutions that were implemented to ensure education.

When there were dossiers [homework assignments], there was no routine, it was just no, I had to pick up the dossiers... I had to hand them in on August 20th and started to do them on the 16th, like 8 dossiers. That was the modality: basically a lot of free time and then ‘okay, I have to do the dossier’. (A. 14)

Adolescents also recognized that their learning was not effective in the online modality, as they lacked opportunities to approach teachers for support on a one-to-one basis: “I could never focus very well at the computer. I always had many questions remaining... my concentration, my learning, was not good” (A. 16). Teachers also reflected on the fact that persisting with online classes faced them with several barriers and challenges. One of them felt they were trying to engage worn-out students. A teacher tried to engage them with “enthusiasm”, but “as weeks pass… months pass… And you continue the same routine, in front of the screen” this became much more difficult (S. 2). Others even had good reasons to doubt the online attendance of students:

It is very likely that some of them were not even behind the screen [with camera off], because when I came to say goodbye to the class, I would say ‘okay, goodbye, leave the meeting’… but I could have the videocall open for an hour longer, and some of them were still online. So that means they were never aware of the class– actually I didn’t know if I was even talking to anyone, because the only thing I saw was symbols and names. (S. 16)

Adolescents referred to a kind of group effect in the online classroom: if they saw that other classmates were not participating in the class, they decided not to participate either. Concretely, they would log in to online classes without really *attending*, which decreased their motivation to engage in learning:

The teacher was there, and well, I couldn’t understand much. I tried anyway. But then, three or four more students were online, and there were forty-something students [in the class altogether]. So, I said ‘if everyone else can do it without classes, then so can I’. (A. 6)

Staff struggled with having a realistic perception of the learning processes and actual progress of their students: “I need to see students’ faces to know how much they understand... I would ask them ‘are we okay?’... and there would be a resounding silence” (S. 16).

In addition to the lack of engagement by students, and consistently with what students reported, the use of the internet and the opportunity to interact with peers during graded assessments allowed for significant increases in students’ average marks that did not reflect actual learning:

And the worst of all was cheating, an enormous amount of cheating. Never in my life had I given so many 7s [maximum grade]... everything was copied... And the school also ordered us to give a 4 [minimum passing grade] even if the student did not hand in anything. So, anyway, everyone passed. (S. 16)

Regardless of the uncertainty, lack of engagement, and challenges of the online delivery of classes, the classes and teaching plans persisted. This reflected the obligation of schools to comply with higher order regulations, but also their determination to continue with “business as usual”, since for many of them state funding is dependent on student attendance: “If there are no students present, there is no subsidy, and no salary, no money, nothing” (S. 2).

Overall, during the pandemic, schools were mostly flexible at a curricular level. The staff members referred to curriculum adaptations, reflected in reduced school hours (longer breaks, shorter classes and school days) and less stringent academic demands (e.g., prioritizing certain subjects above others in line with the recommendations of the Ministry of Education), which also held in the transition to on-site school.

The consequences of the lengthy school closure and persistence with the online curriculum regardless of the learning challenges of adolescents were visible when the students returned to school. Upon their return, adolescents were not sufficiently prepared to continue with their education. Several staff participants recalled students openly claiming that they had not learned anything: “There were two lost years, regardless of online classes” (S. 16).

The kids came back with zero capacities... The ones we received from the 1st year of high school had the personality of 1st year of middle school, or even less. We had to dig from very low... with basic abilities in languages and math. (S. 10)

Staff were aware that starting with regular tests immediately would not work. For example, a “gradual” (S. 16) approach was used, in which short tests were averaged with work group assignments. Sometimes, schools even prohibited the issuing of failing grades.

As a result of the educational strategy implemented during lockdown, teachers and staff became worn out. This was evidenced by a higher number of medical leaves, conflicts between teachers, and emotional outbursts in the return to face-to-face education: “Truly, it [remote teaching] was very draining. There was a point when I couldn’t take it anymore, literally” (S. 11). Moreover, staff recognized that specialized support for teachers was lacking, plausibly contributing to their exhaustion: “The psychological support is for the students, but never for us, despite all we see and have to witness with the students” (S. 16). Strikingly, while many in the school staff community endorsed the need for psychologists, psychologists themselves reflected on the lack of receptiveness from teachers towards mental health initiatives, in the urge to continue with the curricular agenda, despite high levels of occupational exhaustion: “We wanted to fully support the teachers... but what did we receive in return? ‘I don’t want any workshops for myself’, ‘I don’t want emotional support’, ‘I don’t want anything from the psychology team’” (S.11).

The accounts of both adolescents and school staff about their experiences of learning/teaching during the pandemic suggest that adolescents experienced an educational system that seemed to have been implemented with little regard for the hardship it caused to both school staff and students.

### Blurred boundaries

The confinement measures and adaptation of school/work settings to remote mode blurred boundaries in routines, spaces, and roles: between school and home life, between school and the family system, and between the activities and roles within families.

#### Blurred boundaries between school and home life

Depending on the different solutions implemented by schools to deliver education, adolescents experienced different levels of structure and school workload. For some of them, the transitions and points of the daily routine became blurred, such as waking-up time, mealtimes, and activities between online classes. As school *came into* the home, school and personal life intermingled, and young people struggled to separate their routines:

I spent all day on the computer, almost. Because [classes] were from 8 am to 5:30 pm... I had to stay in my pajamas all day because in the morning I would get up just in time to connect. So I took a shower at night. (A. 9)

This lack of boundaries between the school environment and the home environment was also evidenced by the fact that school classes sometimes took place in the adolescent’s bedroom, where they would usually spend their time doing leisure activities:

I sleep on the second floor... I would go downstairs only a few times... I stayed upstairs almost all day. The lockdown caused this, because I had the computer in my room, so I was in my room for all the classes. (A. 2)

Another challenge was studying with other children or parents at home (e.g., having to share reduced spaces and even the same computer to attend the online classes as required). Some adolescents reported noise around the house which made it difficult to focus. They mentioned being distracted by other members of the household (e.g., having to do errands for the family), and even family conflicts happening during online classes.

Overall, the school’s intrusion into the home environment was difficult, as many adolescents had to rearrange their daily routines and working and studying spaces, at the expense of well-defined time and space for their home life.

#### Blurred boundaries between school and the family system

Despite the lack of actual contact between adolescents and their schools, the school “entered” the family system. For example, the educational system made new arrangements for the delivery of food supplies provided by the government for the most economically disadvantaged students. Normally, students would receive breakfast and lunch at school every day. Due to the pandemic, this was no longer possible and grocery boxes were delivered by school staff or picked up by the student when the safety measures allowed for it.

Then I transitioned to a more social role, so to speak... family grocery boxes started to arrive... and I was the one that would deliver them to all the kids on the list. So, for a long time I went to each of their houses. (S. 6)

Additionally, given the crisis, schools decided to provide printed study materials, school clothes (in some cases), and resources to ensure that all students would be able to participate in online schooling, such as tablets and internet connection means.

‘Quickly! tablets!’, so the students could have the devices and tools that would allow them to enter the classes, having the necessary connectivity. It [the connectivity] was strengthened with SIM cards– hundreds of hundreds of SIM cards were delivered... we have kids from rural areas, that come from very remote places, where internet does not reach; otherwise they would have to go to the top of the mountain for access. (S. 2)

This way, the school entered the family system, overstepping the boundaries of its educational role and acting as a social guarantor. The boundaries were also blurred the other way around, from household to school. In a context where teachers were no longer readily and physically available in person, the most proximal source of learning support for adolescents was their family. Family members began to perform an educational role for most of the participants. Parents became the main learning support mentioned by the adolescents: “My dad always says ‘should I hire a teacher so you can understand?’ Or ‘should I help you?’... When I have many assignments piling up, my mum says ‘should I do one for you?’” (A. 1). Support from siblings and other family members was mentioned in fewer cases. Importantly, not all participating adolescents perceived their parents as supportive. They indicated that their parents could be an additional source of stress for their learning, for example, through parental pressure on them to improve their grades under schooling conditions that were already challenging.

#### Blurred boundaries within the family system

Adolescents and their families were compelled to spend more time together and do most of their daily activities at home, given the restrictions. This introduced the challenge of reorganizing family functioning, from the spaces that each family member used at home, to the responsibilities they could individually take. The presence of adolescents at home created opportunities for them to take on some responsibilities and duties at home, such as chores, for example. While some specified that the roles were well distributed among other members of the household (such as siblings), some others reported performing roles that seemed not developmentally appropriate, such as being responsible for the greatest share of household chores or for taking care of younger siblings during the day. Some adolescents even indicated that they had to combine household chores and online classes:

My mum seized the chance of having me home so that I could help her more with stuff like cooking lunch… Never in my life had I cooked lunch or washed the dishes, but [now] I was always tidying up... Always, with the classes in the background. (A. 17)

Some participants described these new roles as something they assumed as a natural part of the new adjustments: “I realized that complaining did not help at all, and that the earlier you finished [chores] the quicker you were free” (A. 7). For some others, the chores were even perceived as beneficial: they described feelings of happiness and purposefulness:

My dad and my mum started working, so I told her [my mum] to leave my sibling with me, at home. Then I was in charge: I looked after my sibling, cleaned up the room, washed, sometimes did practically everything... I did it happily because I was helping my family, and I had something to spend my time on. (A. 6)

There were different experiences on how to combine chores with studying. While for some, chores were perceived as getting in the way of online classes, for others they “helped to stay focused and not distracted” (A. 15).

The disruption of individual activities out of the home (e.g., going out to work or school) also blurred the family structure, by causing sudden changes in the household composition. For example, sometimes grandparents or adult siblings were reincorporated to the household, or visits to parents who did not live at home were interrupted. In some cases, this created changes in the main caregiving figures for children at home.

Adolescents ended up spending most if not all their time at home with family members. In some cases this increase in time spent together led to changes in the quality of relationships. Many adolescents reported an increase in conflict with other family members. For instance, one participant indicated that the continuous presence of his father was highly stressful: “My dad also started working here on the computer and all that stuff... I could not bear him... and my dad was more and more stressed” (A. 2). Given these blurred boundaries, another participant expressed the desire to “live alone in a forest, because I couldn’t bear my mum, or my brother, and dad barely talks” (A. 17).

Other adolescents however indicated that being at home together improved their relationships with their family members. One adolescent specifically mentioned that being at home allowed them to share more and strengthened their relationship:

[…] to accept that we were living through this and we had to move forward together. Then I started to bond more with my dad... With my mum the mother-child relationship was deepened, because I got to speak about many more things with her. (A. 19)

In these ways, the lockdown stirred up the family system. It changed dynamics, roles, and relations, and required quick adaptation from adolescents and family members to cope with the new challenges posed by the pandemic disruptions.

### Development will (try to) find its way

Despite the findings that adolescents were in crisis; support systems often failed; lines between different contexts were blurred; and schools continued their ‘show’, the adolescents showed signs of normative development. Even under mandatory social distancing circumstances, developmental needs and tasks claimed their place in adolescents’ lives. Moreover, adolescents seemed to show a capacity to bounce back in their wellbeing over the year in which normality was restored.

#### Social domain

As adolescents struggled with the fact that their peer relationships were not as usual (broken support system), they also used all available means to remain connected in the social domain. Most adolescents reported staying in touch and making new connections by using social networks. A significant portion of them reported constant digital contact and permanent availability for interaction, particularly those with well-established friendships before the lockdown:

The fact of having 24/7 availability, to call a friend at, I don’t know, 12:00 at night, knowing that he is awake, either to talk for a while, to unwind, maybe to game, something like that. (A. 19)

Looking for other alternatives, like not meeting with my friends, but talking in a call, or sometimes we made like coffee or cooked together while in a call, or I don’t know... we talked a lot, watched movies with my friends. So, it was like searching for other options, like finding a way, so everything would not be so strict. (A. 14)

On the return to face-to-face education, most of the adolescents identified the possibility of having more contact as the greatest benefit of return: talking, going out, seeing each other, being able to meet other people. The return represented a possibility to recover opportunities for socialization that were normative and that the adolescents hoped to reestablish. It is worth noting that the supportive role of family members, especially during the return, was barely mentioned, compared to the emphasis on the importance of friendships and peers: “Meeting again with my classmates, going back to the routine we always had, playing ping pong… every break we have done the same, and it is what I have liked the most, and meeting up again, hanging out again” (A. 13).

A drive for interaction was also evidenced in accounts by school staff. They identified in adolescents a search for socialization: seeking attention, physical contact, and affection from their peers: “They could touch each other, give each other hugs, also with the teachers’’ (S. 17). This can also be seen from the perspective of reestablishing normality. Staff mentioned that they observed the adolescents’ need to socialize and hypothesized that their catching up with well-being over the months after the return was due to the fulfillment of this developmental need, enjoying the freedom “that goes on in normal life” again (S. 13).

Interaction with peers seemed to play a facilitating role in the transition back to normal, particularly for those adolescents presenting issues linked to social anxiety, who felt encouraged by their peers to socialize: “*They* contacted *me*– I wasn’t going to reply, because I felt ashamed, so *they* talked to me again” (A. 17). This supportive role was also reported in relation to readapting to school and learning: “[…] with the help of my best friend. She really helps me to do assignments when I don’t understand something, because she is good at it” (A. 16).

As would happen normally, during the pandemic and beyond adolescents experienced changes in their peer connections and meaningful relationships. Some participants mentioned achieving greater closeness with friends or making new connections during the lockdown and more easily on the return to school. However, difficulties in friendships were also mentioned: one student referred to a reduction in the number of peer relationships, and others reported conflicts that disrupted relationships with best friends, mainly caused by gossip or the involvement of third parties. However, and interestingly, in several cases these disruptions led to new, often more meaningful relationships, implying some reevaluation processes of friendships:

I realized that the people I talked to left me out, that I was actually never necessary in that group... I tried to get closer, but they formed a group apart from me... But the good thing was that I started to share with other people, some classmates I had never spoken to. And I found that there were many more people I shared many points of view with, but I had never shared with them. (A. 19)

I really learned to select my friends better, because there were many times during the pandemic that I realized those were not friends, but rather people to hang out with or to gossip with sometimes, something very superficial... I started to understand how to find deeper friendships. (A. 14)

In general, socialization with peers was a predominant need for adolescent participants and was very much evidenced in the reopening of society. There were only a very few participants for whom remote education was preferable to in-person education (i.e., those with a home-schooling situation implemented even before the pandemic, or adolescents who described social anxiety issues). These students perceived the presence of peers and the school context in general as stressful in the return to normal. However, for the vast majority of adolescents the return to face-to-face interactions with peers constituted an improvement in their connectedness with friends and provided opportunities to establish new relationships (e.g., with romantic partners).

#### Individual domain: personal development

Adolescents sometimes reflected on their personal processes during lockdown and beyond, in a way that showed that they experienced insight into different domains. Several participants described becoming more self-aware, regarding their own behavior, thoughts, and relationships. One participant mentioned, for example, that “being alone” helped them discover themselves better: “I could discover myself... because I know what things make me uncomfortable, I know which things I can say I like, and which ones I don’t” (A. 1).

Another participant mentioned that the pandemic made them “value time better”, moving them to reflect on what was important for them: “I lost a lot of time and now I am making the most of it; I lost two years and now I am living these stages” (A. 14). Some adolescents were able to reflect on the impact that safety measures had had on their self-concept: “It was also a bit hard to take the mask off, have confidence again: this is me, have I changed or not? And I decided to accept myself as I am” (A. 19).

Most adolescents described processes that involved personal growth or reaching “maturity” during the pandemic years, for example by being able to make their own decisions more consciously, even in small daily things, in connection with their need to recover a more normative autonomy. One adolescent for example mentioned getting up earlier so they could pick up a friend on the way to school, and explicitly stated “that is also a decision” (A. 7).

It is worth noting that this overall tendency to adapt was not free of complaints and struggles. For some participants, although they reported benefits of the return, some mental health complaints still persisted at the time of the interviews, and they were still having difficulty adapting to school: “[I still feel like] I will explode any moment in the classroom” (A. 1). In the same vein, although staff reported a perceptible decrease in the seriously concerning behaviors observed in the first months after the return to school, the difficulties had not completely vanished and “kids are still explosive” (S. 5).

However, all things considered, school staff remarked that adolescents in general organically bounced back to more adaptive behavior as the school year went by, with some pointing out a clear contrast between the first half of the year and the second half after the winter break. This conveys the idea that development finds its way thanks to the more normative conditions of the current context: “In the beginning there were many crises [episodes of children presenting difficulties at school], but over time that is fading away” (S. 14); “Even those we thought started really badly– we feel they finished very well” (S. 15).

**Fig. 1:**
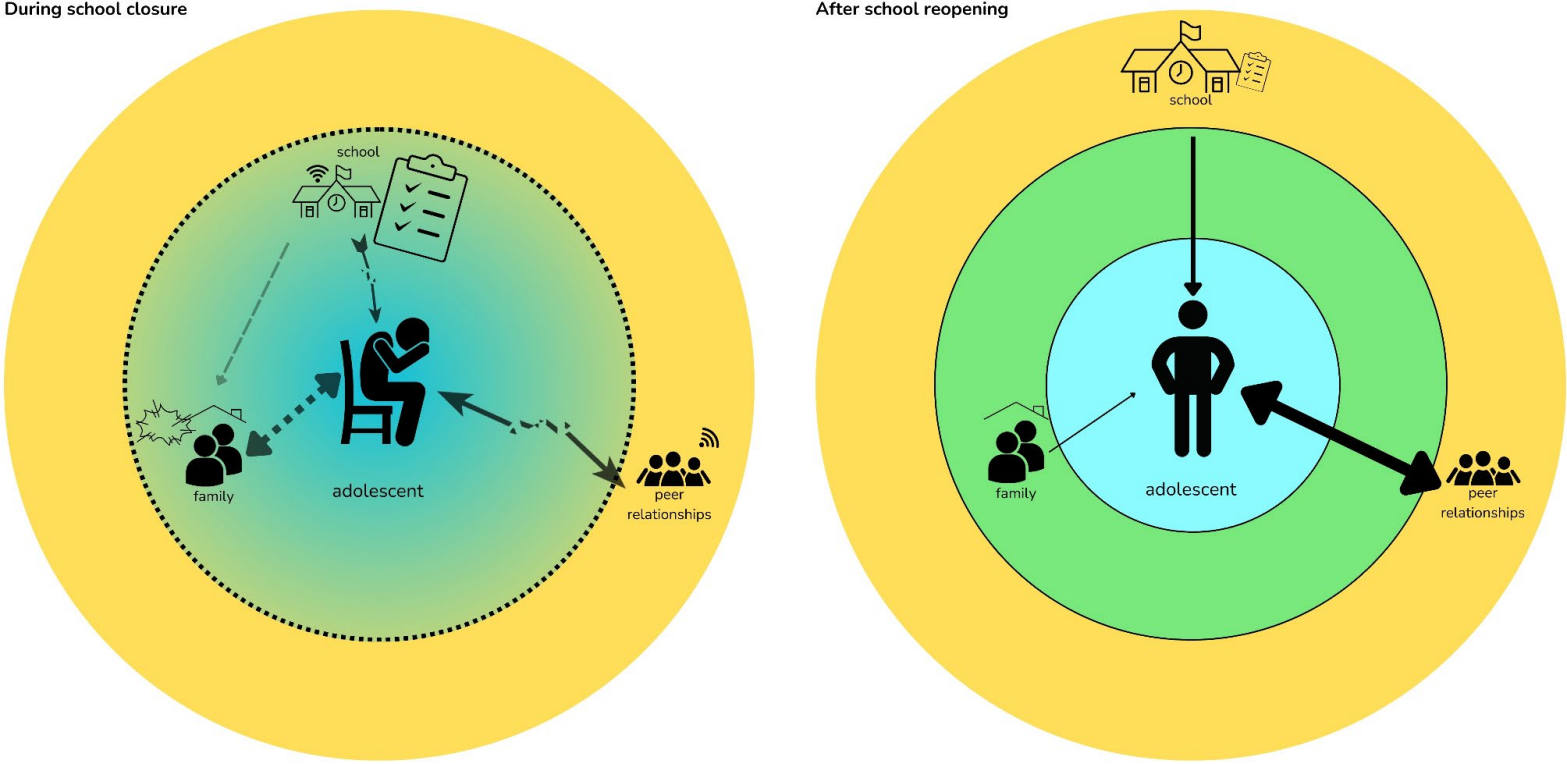
Conceptual summary of themes and school functioning in Chile during the COVID-19 pandemic.

A graphic summary of the findings is presented in Fig. 1.

Figure 1 illustrates the experiences of adolescents during the school closure and in the return to face-to-face schooling, as reported in the themes of our study. The adolescent is placed in the center, surrounded by relevant social systems in different spheres of the environment (home system, school system). Theme 1: The mental health crisis is represented during the school closure with a sitting adolescent, given the conditions offered by the surrounding systems described in themes 2, 3 and 4. Theme 2: “Broken support systems” are represented by broken arrows. Other supports that were present but described as less substantial are represented by dashed arrows. Theme 3: “The school agenda must go on” is represented by a large to-do list during the school closure, decreasing in size during the reopening. Theme 4: “Blurred boundaries” is represented by school, family and youth coexisting in the same space (home system) on the left. The social systems are better differentiated in the reopening. Theme 5: “Development will (try to) find its way” is represented by the change of the adolescents’ mood from left to right, provided that the conditions for normative development gradually improved in the school reopening.

## Discussion

This study aimed to obtain in-depth information on the experiences of Chilean adolescents regarding their mental health during the COVID-19 lockdown and in the transition back to in-person education, and to portray features from ecological systems (family, peers, and school) that played a role in adolescents’ adaptation. Consistent with the extensive global literature detailing the negative impact of the pandemic [15, 18, 49], our results point to a clear emotional toll on Chilean adolescents. Our participants recalled a wide range of complaints in this period, mostly -but not restricted to- internalizing symptoms. Nevertheless, our results also testify to the overall capacity of adolescents to catch up with development and wellbeing once normality is restored.

Several phenomena in the developmental contexts of adolescents were identified in our results as contributors to the mental health crisis during lockdown. First, the lockdown and the protracted closure of schools heavily disrupted adolescents’ connections with their sources of social support. Second, with the lockdown measures, all daily activities “imploded” to the physical space of the house, blurring the boundaries between school and home life, between school and family, and between household members. Third, the school’s focus on curricular goals led to a risk of exhaustion and posed challenges for students’ learning processes.

On the adaptive side, we found (in line with previous research) that the digitalization of friendships helped mitigate feelings of loneliness [50, 51]. This highlights the role of friendships as a buffering factor for adolescents’ wellbeing [9] and as a priority developmental need that found its way even in the face of restrictions. Despite the detrimental effects of overuse of screens and social media during lockdown [52], socialization through media was highly relevant for adolescents’ social and identity development [53].

School would normally be a space to connect not only with peers, but also with other protective adults outside the family network, a function that is especially relevant for adolescents with less supportive households [54]. During the pandemic, school staff faced difficulties in monitoring students’ wellbeing. Moreover, the interviews did not reveal evidence of structural initiatives targeting emotional wellbeing or social connectedness between students. Schools focused rather on the academic curriculum, and had a hard time connecting with students during the pandemic. Consequently, school staff were unable to serve as protective agents for adolescents. Schools’ function as an agent of learning was actually impaired by the insistence on maintaining a school routine that was as normal as possible, even when both students and teachers were becoming worn out. Students were exposed to an unprecedented educational system that likely exceeded their developmental capacities (requiring them, for example, to develop autonomous study methods and time management skills), and overlooked the conditions in which their learning took place (e.g., connectivity issues, lack of access to the teacher, distractions from more people at home). Though the policies were intended to guarantee the universal availability of education, they could not fully cater for fundamental aspects such as accessibility, acceptability from the students, or adaptability to students’ needs. It is therefore questionable whether adolescents’ rights to quality education were actually met [55].

The remote education solutions were received by the students in different ways. As reported by participants, the online contact between teachers and students during this time was limited. Students struggled to find tutoring when needed and easily became disengaged from the learning activities. In some studies, the lack of teacher support during the pandemic has been documented among adolescents as a significant downside of remote education [41]. In our study, by contrast, some participants described remote education as beneficial for both their learning and wellbeing (for example, in a student who had difficulty socializing). This is consistent with other studies reporting a diversity of adolescents’ opinions and views on online schooling [56].

In addition to the lack of in-person socialization during lockdown, adolescents also faced the instability of their social circles in the transition to on-site schooling. Consistent with previous findings, student absenteeism and an increased turnover among teachers were reported as challenges on the return [30], and these factors may have impaired social connectedness and the opportunities to create stable friendships. Consistent with literature reporting a negative impact of the COVID-19 pandemic on youth experiencing school transition [57], our results show that students who were newly enrolled during and immediately after the pandemic faced extra challenges. As they did not have any connections to the school or their peers prior to the remote teaching period, it was particularly difficult for them to connect with their school and peers during lockdown and on the return. Stability of school friendships has been found to predict greater school belonging [58]. Importantly, a sense of belonging at school has been linked to wellbeing among Chilean teenagers during the pandemic [59]. All in all, the evidence calls for more attention for newly enrolled students as a high-risk group.

Next, our results are consistent with emerging qualitative literature in relation to variability and changes in adolescents’ home life and routines [41]. As a result of the disruption of daily life, the lockdown altered relations between school, home, and family (e.g., the incursion of school into home life, and the loss of boundaries in family functioning). The theme “blurred boundaries” that we identified in our study provides concrete support for a pandemic-related reformulation of Bronfenbrenner’s Ecological Theory Model as proposed by Frankel and Sampige [60], in which the exosystem enters the microsystem, blurring the boundaries between the three systems (onto-, micro- and exosystems). The increase in time spent together within the family increased the opportunities for conflicts and led to redefinitions of structure and roles among family members, but also opened up opportunities for more bonding. In many cases, adolescents had to take on different roles at home during school time, as parents also struggled to combine their duties as workers and caregivers. In this context, it could be a challenge to define times and physical spaces for school, work, and leisure. Previous studies in Chile have linked economic disadvantage and overcrowded housing conditions with worse mental health [61]. In the pandemic, a perceived lack of space at home was found to predict anxiety, depression, and stress [62]; given this evidence, it is plausible that students from more disadvantaged families were more severely impacted in their mental health due to blurred boundaries (e.g., lacking proper physical space for study or leisure, or being required to take care of siblings or do other chores during school time).

Adolescents’ emotional problems during the pandemic came to the attention of educators (and of public opinion) more explicitly on the return to on-site schooling. The lack of support systems during lockdown to identify and support adolescents at risk meant that mental health difficulties went undetected. This underscores the role of schools in the prevention of mental health problems when other systems fail to do so, and in taking protective action in the most severe cases.

Despite the mental health crisis observed during lockdown and in the initial stages of the return to on-site schooling, both adolescents and school staff described processes of readjustment and improvement in emotional well-being over the course of the school year, once “normality” was restored. Socialization was identified in our findings as the great benefit of the return, which (in line with previous reports) highlights its role in development [9, 63]. Taken together, our findings suggest that once the balance within and between systems was resumed with the “return to normal”, most individuals bounced back to positive mental health outcomes, aided by the support of peers and the return to regular schooling systems and routines. The general literature on post-disaster adolescent mental health posits that most adolescents show adaptation trajectories of resilience and recovery [64]. However, existing studies regarding mental health trajectories among youth in the course of the COVID-19 pandemic yield heterogeneous findings [65]. For some adolescents, the experience of adversity may limit their ability to capitalize from socialization opportunities, which in turn increases the risk of maladaptive outcomes [66]. Some of our participants indicated more pervasive impairments to mental health on the return; in such cases help may remain necessary beyond the transition to normality.

Given the social conditions in the country [67] and the high incidence of mental health problems prior to the pandemic [34], the Chilean population is at high risk of psychological sequelae as a result of the pandemic. Importantly, socioeconomic status and educational financial grounds are related to the severity of internalizing symptoms and resilient functioning among Chilean adolescents [31]. Therefore, it is plausible that the major social inequity not only impacted the educational system, but also the way adolescents and their families navigated the lack of efficient support and the issues of blurred boundaries involved in the switch to remote education.

### Strengths and limitations

To our knowledge, this is the first study in Chile to report on adolescents’ mental health during and after the pandemic from a qualitative perspective, contributing not only to the understanding of adolescents’ experiences at the local level but also to mental health scientific knowledge at the global level. This study contributes insights on the alterations between and within systems and on the adverse social context that fueled the psychological impact of the pandemic in Chile. We took a retrospective perspective on the crisis, stressors, and changes both during the lockdown and during the return to “normal”, as adolescents regained more agency over their lives. Asking participants to recall a two-year period prior to the interviews is not free of recall bias. Retrospective and prospective reports on adverse experiences moderately agree [68], losing rich details and fluctuations in wellbeing that could only otherwise be collected longitudinally. However, participants were able to report their most significant memories about the period, which were sufficiently elaborate to generate rich data with detailed experiences. Limitations regarding potential recall bias could be improved in future research by addressing experiences in adversity longitudinally, across different stages of community crises. However, this endeavor can be challenging in the early stages of crises when it is ethically difficult to assess adversity. Research could benefit from cohort studies in which the consequences of exposure to adversity can be followed over time, particularly in countries such as Chile, that are frequently exposed to community hazards (e.g. natural disasters).

Another limitation was the low response rate to the school recruitment. Plausibly, for most schools committing to our study towards the last months of the school year would conflict with a compressed school agenda upon the reopening year. Next, we conducted this research in an area where schools receive a constant influx of study invitations from local undergraduate students. Despite the relatively small proportion of participating schools, these had different background characteristics that added to the diversity of our participants. The number of interviews provided us with rich data that shed light on a wide range of experiences of adolescents and staff.

Our analysis did not distinguish between individuals in terms of demographic characteristics such as age, gender, or family composition, and it is not possible to disaggregate the unique impacts on wellbeing of specific aspects of the pandemic experience (e.g., COVID-19 infection, lack of on-site schooling, social restrictions). However, the study does provide a general overview of adolescents’ functioning and the systems in which the adolescent operates. As such, it contributes to generating a complex picture of the pandemic experience, and a better understanding of the situation of young people in the wake of the pandemic.

The results of this work reflect the experiences of adolescents and school staff, from their own, subjective perspectives. Therefore, the results do not necessarily reflect the actual initiatives and efforts undertaken at the school or government level to safeguard mental health during the pandemic. However, the views of students and staff are essential to reveal how these measures were experienced. Next, our results depict the experiences of students and schools in a specific region in Chile. Since every location in Chile underwent dynamic lockdowns on the basis of local health risks and infection rates, the duration of school closure and the decisions regarding reopening differed slightly from one community to another. Consequently, the associated challenges in terms of policies -at the school and city level- and wellbeing may also have differed depending on the location. In addition, this study did not involve participants from fully private schools. This means that it does not include the unique experiences of a different group that may not have experienced the same educational barriers as our participants; students in private schools experienced different educational solutions and may have faced different challenges that are not reflected in our study. For these reasons, our findings are not transferable to other communities with different educational and social realities. Moreover, we did not differentiate our results on the basis of school size or take into account the impact this may have had on the ability of staff to monitor students’ wellbeing on a one-to-one basis and to (re)establish student-teacher relationships. We did our best to include equal numbers of female and male students from the different classrooms and participating schools, as well as including school staff who could contribute different perspectives on adolescents’ wellbeing. In all, our study can throw light on the experiences of adolescents in similar contexts during lockdown.

As researchers, we are aware of how our positionality may have biased our interpretation of data through the lens of our own origin and culture, and of the location where we experienced the pandemic (for most of us, far from the stringent measures in Chile). The context in which Chilean and Dutch adolescents experienced the pandemic differed in several ways (i.e., social inequality, poverty, working/housing conditions, education system). We acknowledge that our experiences and backgrounds differ from those of the vast part of Chilean society, and from those of our participants; this may have biased our understanding of the experiences reported in our study, giving them more or less importance in the context of our own lives and pandemic-related experiences. Additionally, the authors share training in education and developmental psychology, and we are aware that our interpretation of the data may differ from that of a healthcare provider, for example. Nevertheless, our analyses were truthfully built around the discourses of our participants and were rigorously discussed in order to ensure the trustworthiness of our findings.

### Conclusions and implications

Our findings provide support for the emotional toll of the pandemic, the multi-system disruptions to the ecology of adolescents’ development, but also a trend of resilience in the wake of pandemic adversity. Drawing on Bronfenbrenner’s ecological theory [69], our results support the notion that normative development requires balance in the exchanges between the child and their systems, each with a distinctive role and mutually dependent on each other. The pandemic experience came to disrupt this balance, affecting many features in the family, community, and cultural systems that are considered to be resilience factors [70]. The school setting is plausibly the most important context for socialization for adolescents, and for most families it helps organize young people’s routine and supervision. While most of our knowledge of child development is based on normal circumstances, the pandemic served as a unique, global natural experiment on the effects that the disruption of “normal” life has on systems that rely on regular schooling. This is particularly true for countries like Chile that experienced protracted school closures.

The long-term consequences of the pandemic as a major chronic stressor on socio-emotional development are not yet fully understood and should be further investigated in this cohort over time. Adolescents’ developmental milestones that may have been disrupted by the pandemic should be followed up in late adolescence and emerging adulthood, with a focus on mental health outcomes and social functioning. Future research would benefit from expanding to biological markers of chronic exposure to adversities like the pandemic, as they have correlates with (mental) health outcomes. Research should also expand on the effectiveness of policy development in response to crises in order to inform decision making. In addition, studies should address the impact of community emergencies on the family system, as this emerged as a relevant setting in our findings.

Globally, the safety measures often failed to support the best interests of children’s integral development [71]. Studies reviewing COVID-19-related mental health policies in Chile claim that no measures were implemented to specifically safeguard the mental health of children and adolescents [72]. Post-pandemic mental health initiatives in school settings should reinforce the curriculum in emotional education, addressing socioemotional skills that had possibly been affected by the pandemic disruptions (e.g., coping strategies, healthy peer relationships). In the future, the negative consequences of such disruptions, such as learning lags and socioemotional sequelae, should be carefully weighed if and when implementing remote education in response to community emergencies. In the event of future community crises, our findings imply that there are minimum standards that must be guaranteed. Educational policies, in coordination with local governments, should promote contact between children and their school, prioritizing socio-emotional wellbeing over the academic agenda, and facilitating spaces (digital or physical) where young people can meet their social and recreational needs, in order to re-establish as normal as possible conditions for wellbeing. As our participants argued, training in tools for staff to respond to students’ emotional difficulties is warranted and should be ensured as a basis of teacher training. Policies should also address structural conditions that support teachers’ wellbeing in their role as protective adults for children. Early and ongoing screening of students’ emotional wellbeing by schools in coordination with health systems, including effective referral, could ensure timely intervention in mental health crises and prevent longer-term effects. Policy initiatives to address the mental health of young people in emergencies should coordinate the intersectoral efforts from education, welfare, health and crisis management systems. In this way, strategies to manage future crises can ensure the conditions to facilitate adolescents’ development, even in stressful circumstances.

## Data Availability

To preserve the privacy of participants, the transcripts analyzed in this study are not publicly available. The interview script is available upon request.

## Abbreviations

COREQ: Consolidated Criteria for Reporting Qualitative Research
OECD: Organization for Economic Co-operation and Development
A.: Adolescent participant
S.: School staff participant
